# Dental Students’ Awareness, Preparedness and Barriers towards Managing Tobacco-Using Patients—A Cross-Sectional Study

**DOI:** 10.3390/ijerph16101862

**Published:** 2019-05-27

**Authors:** Dave Ching Yeung Liu, Tracy Chui Yi Ho, Duangporn Duangthip, Sherry Shiqian Gao, Edward Chin Man Lo, Chun Hung Chu

**Affiliations:** Faculty of Dentistry, The University of Hong Kong, Hong Kong, China; daveliu@connect.hku.hk (D.C.Y.L.); hochuiyi@connect.hku.hk (T.C.Y.H.); dduang@hku.hk (D.D.); gao1204@hku.hk (S.S.G.); hrdplcm@hku.hk (E.C.M.L.)

**Keywords:** smoking, public health, dental education, nicotine replacement therapy, tobacco cessation counselling

## Abstract

*Aims*: To evaluate Hong Kong dental students’ perceived awareness, preparedness and barriers towards managing tobacco-using patients. *Methods*: A validated questionnaire was administered to dental students who were in their clinical years (the third, fourth, fifth and sixth year of study) in 2017 at the University of Hong Kong. The questionnaire consisted of three sections: (1) awareness towards practicing tobacco cessation counselling (TCC), (2) preparedness in terms of confidence, knowledge and clinical practices when managing tobacco-using patients, and (3) perceived barriers to counselling. *Results*: All 206 invited students had participated this study. Most (93%) agreed that dentists should deliver TCC. However, only around a quarter (26%) of students were well-prepared to help patients in tobacco cessation. While 60% of students agreed nicotine replacement therapy (NRT) was helpful for patients to quit tobacco use, only 28% understood its mechanism of action, and merely 16% were knowledgeable enough to introduce NRT to their patients. Two-thirds (62%) of students felt they did not have sufficient skills at this stage of their training. *Conclusions*: Most Hong Kong dental students had good awareness that dental professionals had an important role to promote tobacco cessation in their patient pools. However, not many of them were well-prepared to manage tobacco-using patients. Common barriers were found to be patients’ apathy and students’ inadequate familiarity with NRT.

## 1. Introduction

Tobacco use is responsible for six million deaths annually in the world [1]. However, these deaths are entirely preventable. There are around 3200 youths who become new tobacco-smokers every day, with an additional 2100 occasional smokers becoming daily smokers [2]. These numbers add up to more than one million youths having their first taste of tobacco every year. Currently, the overall prevalence of tobacco-users in the Hong Kong population is 10.5%; the prevalence in males and females is 19% and 3%, respectively [3]. The government of Hong Kong has been curbing this epidemic with multiple measures by increasing taxation and hence total price on tobacco products and producing advertisement delivering anti-tobacco messages [3]. Tobacco use can cause damage to nearly every system in the body, such as the respiratory system (obstructive pulmonary disease and tuberculosis), cardiovascular system and reproductive system [2]. Moreover, it was well established that tobacco use could increase the risk of cancer that involves the lung, liver and colorectum [2]. In addition, tobacco can significantly influence oral health. Tar within the tobacco can deposit on both hard and soft tissues, resulting in unsightly staining of teeth, whereas the benzopyrenes within the tar components could stimulate melanocytes to produce melanin which causes smokers melanosis, resulting in deeply stained soft tissue. Carbon monoxide causes tissue hypoxia, reduces the salivary flow rate and pH value [4], and destroys the protective macromolecules of saliva [5]. Nicotine, as a vasoconstrictor, reduces blood perfusion and therefore diminishes the supply of nutrients and oxygen to oral tissue. Oral cavities of tobacco users are more prone to delayed wound healing, infection and necrosis [6,7,8]. Tobacco constitutes over 70 known carcinogens [9]. They can be crucial determinants in the oncogenesis of the oral tissues [10]. Studies have also shown that tobacco use can also contribute to halitosis because the amount of volatile sulfur compounds was significantly increased in the periodontal pockets of tobacco-using patients [11]. The heat generated from direct tobacco use causes dilated and inflamed minor salivary gland ducts, which typically affects the hard palate, resulting in nicotinic stomatitis [12]. Moreover, the use of tobacco has a dose-dependent association with the risk of tooth loss [13]. Dental treatment outcomes have been found to be less successful in tobacco-using patients. In the context of periodontology, the treatment outcome of both non-surgical and surgical periodontal therapy was worse in tobacco-users than that in non-tobacco-users [14]. Moreover, tobacco use was a risk factor associated with the higher failure rate of dental implants [14]. Also, in patients who are victims of oral cancers, continued use of tobacco after surgery was found to be related to increased risk of relapse and development of a second primary tumor [15,16].

Many studies showed that even brief or simple counselling provided by health professionals could substantially boost the quit rates of tobacco-using patients [17,18,19,20,21]. Dentists were reported to be ‘uniquely and advantageously positioned’ to provide tobacco cessation counselling (TCC) [22]. Researchers have found that 59% of the patients who attend a dental clinic expected the dentists to provide tobacco-cessation services regularly [23]. Only 3% of patients can quit tobacco use solely by their willpower, i.e., can accomplish the tobacco cessation without any assistance such as counselling and pharmacotherapy. It was reported that with three minutes of advice from healthcare professionals, another 2% of tobacco-smokers would quit smoking; and with 10 minutes of advice, another 6% would cease consumption [24]. When dentists provide long-term TCC, up to 18% of tobacco-using patients would quit [20,21]. Thus, the role of the dental team in delivering tobacco cessation advice is of great significance. Pharmacotherapy has been reported as a useful treatment item; it was shown that the use of nicotine replacement therapy (NRT) alone can already assist 28% of patients to quit smoking [25]. However, research has shown that in Germany, NRT products could be purchased over-the-counter by the public without prescription from healthcare professionals; and the results showed that around one-third of the customers purchased NRT products at the wrong dosage; or worse, some patients purchased NRT products without knowing that they are actually contraindicated for NRT [25]. Meanwhile, only 63% of the patients complied with the directions for use [25]. As the combination of counselling and pharmacotherapy were found to be more effective than either alone, dentists were recommended to provide both TCC and NRT for their tobacco-using patient [18].

According to the Clinical Practice Guideline for Treating Tobacco Use and Dependence, clinicians are responsible for providing repeated interventions, documenting tobacco use status and providing brief cessation counselling [18]. In Hong Kong, it was found that 77% of medical doctors documented patients’ tobacco usage history, but only 29% of the doctors took up the role of providing tobacco cessation advice [26]. As for dentists, it was reported that only 38% of them had assisted patients in tobacco cessation [27]. These results showed the situation in Hong Kong was worse than the situation in United Kingdom (UK), where 68% of the UK dentists and 75% of Northern Irish dentists felt obligated to provide tobacco cessation services and provided information on tobacco cessation for their patients [28,29]. Tobacco education and tobacco cessation interventions should be included as a crucial component of undergraduate dental training. Up until now, there is limited information regarding Hong Kong dental students’ attitude and practices towards tobacco cessation for their patients. The aim of this study is to evaluate Hong Kong dental students’ awareness, preparedness and perceived barriers towards managing tobacco-using patients, and to compare the responses between students in different years of their study. The null hypothesis was there would be no difference of awareness, preparedness and perceived barriers towards managing tobacco-using patients between students at different years of their dental study.

## 2. Materials and Methods

A cross-sectional questionnaire study was conducted in Prince Philip Dental Hospital in Hong Kong (Supplementary Materials). The Faculty of Dentistry at the University of Hong Kong is the only dental school in Hong Kong. Dental students need to study for six years in the Faculty of Dentistry at the University of Hong Kong to obtain the bachelor degree of dental surgery. All dental students who were in their clinical years (third, fourth, fifth and final year) were included in this study. No specific exclusion criteria were set. Ethical approval was obtained from the Institutional Review Board of the University of Hong Kong/Hospital Authority Hong Kong West Cluster (approved No.: UW 17-226).

The questionnaire was partly based on an earlier study by Murugaboopathy et al. [22], and additional items were developed. A pilot study was conducted in early February 2017 and revisions were made as necessary. The students who participated in the pilot study were not included in the final sample. The questionnaire was distributed in both electronic and paper format. The survey had been distributed to participating dental students in February and March 2017. The questionnaire consisted of three main sections: awareness towards practicing TCC, and preparedness in term of confidence, knowledge and clinical practices when managing tobacco-using patients, and perceived barriers to counselling. Twenty-three questions with a five-point Likert response scale, ranging from strongly agree to strongly disagree, were used to investigate the awareness and preparedness regarding the confidence and knowledge towards dental professionals’ role in TCC and perceived barriers to counselling. Eighteen questions with yes/no choices asked about dental students’ awareness on professional responsibility items and preparedness regarding the clinical practices towards TCC for students with/without tobacco-using patients. Another four items asked students if TCC is unnecessary, should be carried out, or complete tobacco cessation must be achieved before commencing dental treatments. The final section contained questions related to demographic data. Students were also asked to report their family and their tobacco use history, which was recorded as non-users, former users, and current users. The surveys were kept anonymous. Therefore, missing data could not be chased after the surveys being collected.

The answers were coded, and data entry was completed with Microsoft Excel. Data analysis was conducted with IBM Statistical Package for the Social Sciences version 23.0 (SPSS Inc., Chicago, IL, USA). Descriptive statistics were generated for all questions. Chi-square tests were used for statistical analysis. Because there were four comparable groups, the statistical significance level of all tests was set at 0.008 (0.05/6).

## 3. Results

The response rate was 100% (206/206). Respondents were 45% male and 55% female. Dental students’ demographic profile, their families’ and their tobacco use status is shown in Table 1. The proportion of dental students being former users and current users were 0.5% and 1% respectively.

### 3.1. Students’ Awareness, Preparedness and Perceived Barriers towards Practicing TCC

The distribution of dental students’ awareness, preparedness and perceived barriers towards practicing TCC in a five-point Likert response scale is shown in Table 2. Almost all (96%) of the students reported they would advise patients to quit tobacco use in their future career life. However, only 60% of students agreed that NRT is helpful for the patient to discontinue tobacco use. Nearly 85% of students were confident in explaining the negative impacts of tobacco use on oral health. Around half of the students (52%) were confident in providing tobacco users with written information to assist their quitting. Only around a quarter (26%) of students claimed to be well-prepared towards delivering TCC. Almost all respondents (96%) reported that they understood the role of tobacco in the etiology of oral cancer. However, only 30% knew about the tobacco cessation counselling protocol. Although nearly 60% of students agreed that NRT is helpful, only 28% of students knew the mechanism of NRT and only 16% of students reported they were knowledgeable enough to introduce NRT to their patients. Patient-related barriers included: (1) they do not have the motivation to quit tobacco use (81%), (2) they will consider tobacco cessation only when they have a related health problem (58%), and (3) they are not expecting tobacco cessation counselling from a dental student (55%). On the other hand, 41% of the students thought that their patients did not listen to them when they were providing TCC for the patients. Students also reported several barriers of delivering TCC. Most of the students (62%) felt that they did not have sufficient skills to provide TCC. Half of the students reported they did not have enough clinical time to introduce TCC to the patients. Some of the students (41%) had concerns about the dentist–patient relationship if they insisted on providing TCC to their patients. In addition, the students reported that there was a lack of tobacco cessation information in the dental hospital (54%), and they were not aware of the existence of a referral pathway (38%).

### 3.2. Students’ Awareness on Professional Responsibility Items and Clinical Practices towards TCC

Responses to items related to professional responsibility and actions that dentists should take when treating tobacco-using patients are shown in Table 3.

Almost all students (93%) suggested that dentists should take action: providing tobacco cessation advice verbally (99%), distributing leaflets or pamphlets (92%) and giving out the hotline of the Tobacco Control Office (89%). However, only 24% of students thought that dentists should prescribe NRT. While almost all students (96%) stated that they would enquire about tobacco usage histories from their patients, around half (49%) of the students had the habit of updating a patient’s tobacco usage history regularly throughout the whole course of treatment. Most students (79%) had patients who were tobacco users. Among this group of students, 91% documented the amount of tobacco use of their patients, but only 29% recorded the types of tobacco used by their patients. Most (85%) of them had made efforts assisting patients to quit tobacco use, but only 42% performed active TCC regularly throughout the whole course of treatment. Less than half (43%) of the students had succeeded in helping a patient to reduce tobacco consumption, and merely 13% had managed to help a patient to quit tobacco use completely.

### 3.3. Variations in Awareness, Preparedness and Perceived Barriers among Different Years of Dental Students

There were statistically significant differences in some items of awareness, preparedness and perceived barriers among different years (Table 4).

### 3.4. The Need of TCC and Quitting Tobacco Use among Different Years

Regarding views towards the need for TCC and quitting tobacco use before dental treatments (Table 5), most of the students believed that TCC should be carried out before treatment for gingivitis (74%), periodontitis (60%), and cosmetic dental treatment (68%). Also, 58% of students agreed that complete tobacco cessation must be achieved before treatments for implant placement. A higher proportion of senior students agreed that TCC should be carried out before periodontal treatments (*p* = 0.013) and implant placement (*p* = 0.004), while more junior students had a higher standard and thought that complete tobacco cessation must be achieved for these treatments.

## 4. Discussion

In this study, most students were found to have positive attitudes and views on the dental professional’s role in managing patients who are tobacco users. Also, it was identified that most students recorded the amount of tobacco consumption of their patients. This could be explained by the fact that in the curriculum of the second year, students were taught to record the tobacco usage history. During clinical sessions, students were expected to report whether the patient was a tobacco user and the amount of tobacco consumption in pack-years. However, significantly fewer students would record the type of tobacco that the patient consumes, and a minority of student would perform continuous TCC. This could further be explained by the fact that even though dental students had early clinical exposure and started treating patients from the second semester of the second year, there was no organized teaching regarding the long-term management of tobacco-using patients. Also, not all students had patients who are tobacco users, therefore some students would not have hands-on experience tobacco cessation interventions. During briefing and de-briefing of clinical sessions, clinical tutors might mention the relationship between patients’ tobacco use and the respective management. However, students from different clinical groups might have learnt differently because different clinical tutors could have different preferences or focuses in the tutorial. Our study also showed that there was an unmet need for TCC education for dental students. Currently, teaching remained at a knowledge-based level regarding the association between tobacco use and oral health. Within the six years of study, students had only one opportunity to attend a motivational interviewing workshop and learn about persuading techniques including giving tobacco cessation advice to tobacco-using patients. There was no problem-based learning nor lecture on how to fully utilize the TCC protocol and NRT in clinical practice. In addition, tobacco cessation was not part of the patient’s clinical treatment plan.

A considerable proportion of the students were not confident nor knowledgeable to prescribe NRT and carry out the effective long-term TCC. In addition, more than half of the respondents underrated the importance of dental professionals in helping tobacco-using patients. Dental students might not understand what an important role they could take in helping patients to quit tobacco use. Together with the lack of teaching of long term TCC and the use of NRT, it could reflect the fact that most of the dental students only took patients’ tobacco-usage history at the first appointment and did minimal verbal advice at that moment. Without long term TCC and NRT prescription, it was apparent why the quit rate of tobacco-using patients was low. To overcome the lack of competence, workshops or seminars should be designed to educate the students the correct indication, dosage, side-effects of NRT, altogether with revision of the 5A model [18] and the TCC protocol [22]. The greatest obstacle which students reported was the lack of patient motivation to quit tobacco use; surprisingly, 81% of students believed their patients were not motivated. However, research had reported that 59% of patients who visited dentists had expected the dentists to help them quit tobacco use [23]. This showed a great discrepancy between students’ perceived motivation of their patients and actual patient motivation. In order to tackle this disparity, emphasis could be placed on reinforcing the idea that there are more motivated patients than the students perceived. The “assess” from the 5A model could also be taught to students in a more in-depth manner to correctly screen patients who are willing to quit tobacco use more precisely. About two-fifths of students thought TCC was ineffective in changing patients’ behaviors towards tobacco use. The students themselves might not be well-prepared or confident to deliver the messages.

According to the Smoking Cessation in a Primary Care Setting published by the government in 2018 [30], one of the most important aspects of tobacco-use cessation programs is counselling provided by healthcare professionals. Dentists have the responsibility to give tobacco-using cessation advice and prescribe NRT to the patients. Therefore, it is important to improve dental students’ awareness and preparedness. The dental school should promote the importance of dental professionals in helping patients to quit tobacco use. A teaching course of tobacco use cessation should be added into the curriculum, which should include knowledge-based lectures, problem-based learning and clinical practices. The TCC protocol and NRT regime should be added into the undergraduate training programs to improve students’ confidence and knowledge. Other strategies, such as developing stickers and re-evaluation charts in patient folders, can help dental students to perform a long-term follow-up of the patients’ tobacco use status. The success of the promotion of TCC and NRT needs collaborations between different institutions. Building a referral system and seeking financial support for NRT are aspects that can be considered to enhance the adoption of TCC and NRT by dental students. In Hong Kong, there is currently little funding provided to the dental school to support any activities which could promote tobacco cessation at the student level, and the collaboration among different institutes has great room for improvement. Without pharmacotherapy products, dental students could find it difficult to provide real assistance to their patients, even if their patients were motivated to use NRT. If strategies could be deployed at the institutional level, not only would dental students receive greater support, but the tobacco-using patients in the dental hospital would also be able to receive holistic care. With the joint effort, future dentists will be able to increase the success rate of dental treatments, and dental patients could be motivated to quit the consumption of tobacco products.

There were some limitations in conducting this research. As a questionnaire was used for data collection, the tendency of respondents to provide positive, favorable responses might be a source of bias, and this might result in an over-optimistic estimate of the attitude and preparedness of dental students towards TCC and TCC implementation. In this study, only 1.5% of students are current or former tobacco-users, which is lower than many other countries, for example, United States (4–43%), Ireland (12–20%), and Greece (47%) [31,32], and the influence of the tobacco-using status of students towards attitudes cannot be accurately analyzed. In addition, apart from sex, study year and personal tobacco-using history, no other demographic information were investigated in this study. In-depth interviews may be considered in the future studies to provide more insightful information. Nevertheless, it is hoped that this study could provide useful information about the current situation of how dental students in Hong Kong are managing their tobacco-using patients and provide insights on designing and implementing future education programs for dental students. To overcome the obstacles mentioned above, there exists a need to integrate additional education programs for dental students on patient motivation, long-term TCC and the correct prescription of pharmacotherapy into the undergraduate dental curriculum across all years to improve the long-term management of tobacco-using patients.

## 5. Conclusions

While most Hong Kong dental students had positive attitudes towards the dental professional’s role in promoting tobacco cessation, not many of them were well-prepared to manage tobacco-using patients. Common barriers were found to be patients’ apathy and students’ inadequate familiarity with nicotine replacement intervention.

## Figures and Tables

**Table 1:** Students’ demographic profile, their families’ and their tobacco use status (*n* = 206).

Students’ Profile	No. (%)
Year of study	
Year 3	51 (24.8%)
Year 4	51 (24.8%)
Year 5	49 (23.8%)
Final Year	55 (26.4%)
Gender	
Male	92 (44.7%)
Female	114 (55.3%)
Respondent tobacco use status	
Non-users	203 (98.5%)
Former user	1 (0.5%)
Current user	2 (1.0%)
Family tobacco use status	
Non-users	154 (74.8%)
Former user	17 (8.3%)
Current user	35 (17.0%)

**Table 2:** Dental students’ awareness, preparedness and perceived barriers towards practicing tobacco cessation counselling (TCC). NRT = nicotine replacement therapy.

Item	Strongly Agree	Agree	Neutral	Disagree	Strongly Disagree
**Awareness**					
I do not consider TCC part of the dentist’s professional role (*n* = 205)	5 (2.4%)	14 (6.8%)	38 (18.5%)	126 (61.5%)	22 (10.7%)
Giving TCC is not part of my role as a dental student (*n* = 205)	3 (1.5%)	20 (9.8%)	19 (9.3%)	135 (65.9%)	28 (13.7%)
I will advise patients to quit tobacco use in my future career (*n* = 206)	92 (44.7%)	105 (50.9%)	9 (4.4%)	1 (0.5%)	0 (0.0%)
I believe TCC by dentists could assist patients to quit smoking (*n* = 205)	18 (8.8%)	95 (46.3%)	72 (35.1%)	18 (8.8%)	2 (1.0%)
I believe NRT is helpful for patient to quit tobacco use (*n* = 206)	10 (4.9%)	113 (54.9%)	72 (35.0%)	10 (4.9%)	1 (0.5%)
**Preparedness**					
I am confident in explaining the negative impacts of tobacco usage (*n* = 206)	25 (12.1%)	150 (72.8%)	25 (12.1%)	6 (2.9%)	0 (0.0%)
I am confident in assisting tobacco users to quit with written information (*n* = 206)	12 (5.8%)	96 (46.6%)	77 37.4%)	20 (9.7%)	1 (0.5%)
I am well-prepared to help patients in tobacco cessation (*n* = 206)	4 (1.9%)	49 (23.8%)	96 (6.6%)	55 (26.7%)	2 (1.0%)
I understand tobacco has a role in the etiology of oral cancer (*n* = 206)	84 (40.8%)	114 (55.3%)	6 (2.9%)	2 (1.0%)	0 (0.0%)
I know what the tobacco cessation protocol is (*n* = 205)	3 (1.5%)	59 (28.8%)	68 (33.2%)	69 (33.7%)	6 (2.9%)
I know the mechanism of action of NRT (*n* = 206)	5 (2.4%)	53 (25.7%)	77 (37.4%)	65 (31.6%)	6 (2.9%)
I am knowledgeable enough to introduce NRT for my patients (*n* = 206)	4 (1.9%)	28 (13.6%)	71 (34.5%)	92 (44.7%)	11 (5.3%)
**Perceived barriers**					
*Patient factors*					
Patients do not expect TCC from a dental student (*n* = 206)	21 (10.2%)	92 (44.7%)	61 (29.6%)	28 (13.6%)	4 (1.9%)
TCC is ineffective unless the patient has a related health problem (*n* = 206)	27 (13.1%)	93 (45.1%)	48 (23.3%)	33 (16.0%)	5 (2.4%)
Many tobacco-using patients do not have the motivation to quit (*n* = 204)	34 (16.7%)	131 (64.2%)	27 (13.2%)	12 (5.9%)	0 (0.0%)
Patients do not listen to dental students during TCC (*n* = 205)	19 (9.3%)	65 (31.7%)	81 (39.5%)	37 (18.0%)	3 (1.5%)
*Operator factors*					
I do not have sufficient skills to provide TCC at this stage (*n* = 206)	17 (8.3%)	111 (53.9%)	58 (28.2%)	18 (8.7%)	2 (1.0%)
I cannot determine a patient’s smoking history without being intrusive (*n* = 203)	6 (3.0%)	44 (21.7%)	78 (38.4%)	71 (35.0%)	4 (2.0%)
I am concerned that the message of TCC may alienate patients (*n* = 204)	6 (2.9%)	51 (25.0%)	86 (42.2%)	59 (28.9%)	2 (1.0%)
Giving unwanted TCC may upset the dentist-patient relationship (*n* = 206)	10 (4.9%)	74 (35.9%)	61 (29.6%)	55 (26.7%)	6 (2.9%)
Clinical time is too limited to do counselling (*n* = 205)	14 (6.8%)	89 (43.4%)	61 (29.8%)	38 (18.5%)	3 (1.5%)
*Other factors*					
There is no tobacco cessation information available in the hospital (*n* = 206)	25 (12.1%)	87 (42.2%)	55 (26.7%)	32 (15.5%)	7 (3.4%)
There is no referral pathway for tobacco-using patients (*n* = 206)	15 (7.3%)	64 (31.1%)	68 (33.0%)	55 (26.7%)	4 (1.9%)

**Table 3:** Dental students’ awareness on professional responsibility items and clinical practices towards tobacco cessation counselling (TCC).

Item	No. (%) of ‘Yes’
**Awareness: professional responsibility**	
No tobacco cessation counselling is needed (*n* = 203)	14 (6.9%)
Giving tobacco cessation advice verbally (*n* = 203)	201 (99.0%)
Distributing leaflets or pamphlets (*n* = 203)	187 (92.1%)
Giving out the hotline for the Tobacco Control Office (*n* = 203)	181 (89.2%)
Writing a referral letter to the Tobacco Control Office (*n* = 203)	138 (68.0%)
Prescribing nicotine replacement therapy (*n* = 200)	47 (23.5%)
**Preparedness: clinical practices of all students**	
I take tobacco usage histories from all patients (*n* = 206)	198 (96.1%)
I ask patients about their tobacco usage status at the first appointment (*n* = 206)	206 (100.0%)
I always document patient tobacco use history in patient folders (*n* = 206)	192 (93.2%)
I update patient smoking history regularly throughout the whole course of treatment for every patient (*n* = 206)	101 (49.0%)
I have patients who are tobacco users (*n* = 206)	162 (78.6%)
**Preparedness: clinical practices of students with tobacco-using patients**	
I only mark down patient as a “tobacco user” in the patient folder for tobacco-using patients (*n* = 163)	9 (5.5%)
I have recorded the amount of tobacco my patients used over the years (e.g., pack year) in the patient folder (*n* = 163)	148 (90.8%)
I have recorded the type of tobacco in the patient folder (e.g., cigarettes, cigars, e-cigarettes) (*n* = 160)	47 (29.4%)
I have made an effort to assist a patient to quit tobacco use (*n* = 160)	138 (85.2%)
I do active TCC regularly throughout the whole course of treatment for every patient (*n* = 162)	68 (42.0%)
I have succeeded in helping a patient to reduce tobacco consumption (*n* = 163)	70 (42.9%)
I have succeeded in helping a patient to quit tobacco use completely (*n* = 160)	20 (12.5%)

**Table 4:** Variations of awareness, preparedness and perceived barriers towards tobacco cessation counselling (TCC) of Hong Kong dental students.

Item	Year 3	Year 4	Year 5	Year 6	Years 3–6	*p*-Value
	Proportion (%) of ‘Yes’ or Positive Response					
**Awareness**						
I will advise patients to quit smoking in the future	49/51 (96.1%)	51/51 (100%)	44/49 (89.8%)	53/55 (96.4%)	197/206 (95.6%)	0.046
No TCC is needed	1/50 (2.0%)	1/50 (2.0%)	1/48 (2.1%)	11/55 (20.0%)	14/203 (6.9%)	<0.001
Giving TCC is not part of my role as a dental student	9/51 (17.7%)	1/50 (2.0%)	2/49 (4.1%)	11/55 (20.0%)	23/205 (11.2%)	0.014
**Preparedness: clinical practices of all students**						
I understand tobacco has a role in the etiology of oral cancer	48/51 (94.1%)	49/51 (96.1%)	47/49 (96.0%)	54/55 (98.2%)	198/206 (96.1%)	0.010
I am well-prepared to help patients in tobacco cessation	11/51 (21.6%)	13/51 (25.5%)	13/49 (26.5%)	19/55 (34.5%)	56/206 (27.2%)	0.032
I update patient smoking history regularly	16/51 (31.4%)	28/51 (54.9%)	25/49 (51.0%)	32/55 (58.2%)	101/206 (49.0%)	0.029
I have patients who are tobacco users	34/51 (66.7%)	35/51 (68.6%)	43/49 (87.8%)	50/55 (90.9%)	162/206 (78.6%)	0.002
**Preparedness: clinical practices of students with patients with tobacco-using patients**						
I have made efforts to assist a patient to quit tobacco use	28/33 (84.8%)	29/36 (80.6%)	35/43 (81.4%)	46/50 (92.0%)	138/162 (85.2%)	0.008
I do active TCC regularly throughout the whole course	13/33 (39.4%)	19/36 (52.8%)	16/43 (37.2%)	20/50 (40%)	68/162 (42.0%)	0.012
I have succeeded in helping a patient reduce tobacco use	9/34 (26.5%)	17/36 (47.2%)	17/43 (39.5%)	27/50 (54.0%)	70/163 (42.9%)	0.002
I have succeeded in helping a patient to quit completely	5/32 (15.6%)	3/35 (8.6%)	4/43 (9.3%)	8/50 (16%)	20/160 (12.5%)	0.011
**Perceived barriers**						
Patients do not listen to dental students during TCC	27/51 (52.9%)	14/51 (28.0%)	25/49 (51.0%)	18/55 (32.7%)	84/206 (41.0%)	0.014
Giving unwanted TCC may upset the relationship	32/51 (62.7%)	15/51 (29.5%)	20/49 (40.8%)	17/55 (30.9%)	84/206 (40.8%)	0.005
The message of TCC may alienate the relationship with patients	22/51 (43.2%)	9/51 (17.6%)	14/48 (29.2%)	12/54 (22.2%)	57/204 (27.9%)	0.010

**Table 5:** Views of Hong Kong dental students toward the need of tobacco cessation counselling (TCC) and quitting tobacco use before dental treatments (*n* = 206).

Item	Year 3	Year 4	Year 5	Year 6	Years 3–6	*p*-Value
**Treatment for gingivitis**						0.083
TCC is not necessary	10 (19.6%)	3 (5.9%)	8 (16.3%)	6 (10.9%)	27 (13.1%)	
TCC should be carried out	30 (58.8%)	43 (84.3%)	36 (73.5%)	44 (80.0%)	153 (74.3%)	
Complete tobacco cessation	11 (21.6%)	5 (9.8%)	5 (10.2%)	5 (9.1%)	26 (12.6%)	
**Treatment for periodontitis**						0.013
TCC is not necessary	0 (0.0%)	0 (0.0%)	3 (6.1%)	1 (1.8%)	4 (1.9%)	
TCC should be carried out	25 (49.0%)	27 (52.9%)	33 (67.3%)	39 (70.9%)	124 (60.2%)	
Complete tobacco cessation	26 (51.0%)	24 (47.1%)	13 (26.5%)	15 (27.3%)	78 (37.9%)	
**Implant placement**						0.004
TCC is not necessary	0 (0.0%)	0 (0.0%)	1 (2.0%)	1 (1.8%)	2 (1.0%)	
TCC should be carried out	20 (39.2%)	10 (19.6%)	22 (44.9%)	31 (56.4%)	83 (40.3%)	
Complete tobacco cessation	31 (60.8%)	41 (80.4%)	26 (53.1%)	23 (41.8%)	121 (58.7%)	
**Cosmetic dental treatments**						0.157
TCC is not necessary	3 (5.9%)	1 (2.0%)	4 (8.2%)	4 (7.3%)	12 (5.8%)	
TCC should be carried out	30 (58.8%)	33 (64.7%)	36 (73.5%)	42 (76.4%)	141 (68.4%)	
Complete tobacco cessation	18 (35.3%)	17 (33.3%)	9 (18.4%)	9 (16.4%)	53 (25.7%)	

## Supplementary Materials

The following are available online at https://www.mdpi.com/1660-4601/16/10/1862/s1, S1: STROBE Statement—Checklist of items that should be included in reports of cross-sectional studies and Pilot test of the questionnaire.

## References

[1] World Health Organization WHO Global Report on Trends in Prevalence of Tobacco Smoking 2015. http://www.who.int/iris/handle/10665/156262.

[2] U.S. Department of Health and Human Services (2014). The Health Consequences of Smoking—50 Years of Progress: A Report of the Surgeon General.

[3] Census and Statistics Department, Hong Kong Special Administrative Region Thematic Household Survey Report No. 59. http://www.statistics.gov.hk/pub/B11302592016XXXXB0100.pdf.

[4] Singh M., Ingle N.A., Kaur N., Yadav P., Ingle E. (2015). Effect of long-term smoking on salivary flow rate and salivary pH. J. Indian Assoc. Public Health Dent..

[5] Petrušić N., Posavac M., Sabol I., Mravak-Stipetić M. (2015). The effect of tobacco smoking on salivation. Acta Stomatol. Croat..

[6] Sorensen L.T., Horby J., Friis E., Pilsgaard B., Jorgensen T. (2002). Smoking as a risk factor for wound healing and infection in breast cancer surgery. Eur. J. Surg. Oncol..

[7] Cobb T.K., Gabrielsen T.A., Campbell D.C., Wallrichs S.L., Ilstrup D.M. (1994). Cigarette smoking and nonunion after ankle arthrodesis. Foot Ankle. Int..

[8] Lind J., Kramhøft M., Bødtker S. (1991). The influence of smoking on complications after primary amputations of the lower extremity. Clin. Orthop. Relat. Res..

[9] U.S. Food and Drug Administration (2012). Harmful and potentially harmful constituents in tobacco products and tobacco smoke; established list. Fed. Regist..

[10] Wynder L., Wynder E.L., Bross I.J., Feldman R.M. (1957). A study of the etiologial factors in cancer of the mouth. Cancer.

[11] Khaira N., Palmer R.M., Wilson R.F., Scott D.A., Wade W.G. (2000). Production of volatile sulphur compounds in diseased periodontal pockets is significantly increased in smokers. Oral Dis..

[12] Taybos G. (2003). Oral changes associated with tobacco use. Am. J. Med. Sci..

[13] Carson S.J., Burns J. (2016). Impact of smoking on tooth loss in adults. Evid. Based Dent..

[14] Twito D., Sade P. (2014). The effect of cigarette smoking habits on the outcome of dental implant treatment. Peer J..

[15] van der Waal I., de Bree R. (2010). Second primary tumours in oral cancer. Oral Oncol..

[16] Vladimirov B.S., Schiodt M. (2009). The effect of quitting smoking on the risk of unfavorable events after surgical treatment of oral potentially malignant lesions. Int. J. Oral Maxillofac. Surg..

[17] Carr A.B., Ebbert J. (2006). Interventions for tobacco cessation in the dental setting. Cochrane Database Syst. Rev..

[18] Fiore M.C. (2008). A clinical practice guideline for treating tobacco use and dependence: 2008 update. A USA Public Health Service report. Am. J. Prev. Med..

[19] Lancaster T., Stead L., Silagy C., Sowden A. (2002). Effectiveness of interventions to help people stop smoking: Findings from the Cochrane Library. BMJ Clin. Res. Ed..

[20] Tomar S.L. (2001). Dentistry’s role in tobacco control. J. Am. Dent. Assoc..

[21] Warnakulasuriya S. (2002). Effectiveness of tobacco counseling in the dental office. J. Dent. Educ..

[22] Murugaboopathy V., Ankola A.V., Hebbal M., Sharma R. (2013). Indian dental students’ attitudes and practices regarding tobacco cessation counseling. J. Dent. Educ..

[23] Campbell H.S., Sletten M., Petty T. (1999). Patient perceptions of tobacco cessation services in dental offices. J. Am. Dent. Assoc..

[24] Cawson R.A., Odell E.W. (2008). Cawson’s Essentials of Oral Pathology and Oral Medicine.

[25] Hasford J., Fagerstrom K.O., Haustein K.O. (2003). A naturalistic cohort study on effectiveness, safety and usage pattern of an over-the-counter nicotine patch: cohort study on smoking cessation. Eur. J. Clin. Pharmacol..

[26] Abdullah A.S., Rahman A.M., Suen C.W., Wing L.S., Ling L.W., Mei L.Y., Tat L.C., Tai M.N., Wing T.N., Yuen W.T. (2006). Investigation of Hong Kong doctors’ current knowledge, beliefs, attitudes, confidence and practices: implications for the treatment of tobacco dependency. J. Chin. Med. Assoc..

[27] Lu H., Wong M.C., Chan K. (2011). Perspectives of the dentists on smoking cessation in Hong Kong. Hong Kong J. Dent..

[28] Johnson N.W., Lowe J.C., Warnakulasuriya K.A.A.S. (2006). Tobacco cessation activities of UK dentists in primary care: Signs of improvement. Br. Dent. J..

[29] McCartan B., McCreary C., Healy C. (2008). Attitudes of Irish dental, dental hygiene and dental nursing students and newly qualified practitioners to tobacco use cessation: A national survey. Eur. J. Dent. Educ..

[30] Primary Care Smoking Cessation in Primary Care Settings. https://www.pco.gov.hk/english/resource/files/Module_on_Smoking_Cessation.pdf.

[31] Smith D.R., Leggat P.A. (2007). An international review of tobacco smoking among dental students in 19 countries. Int. Dent. J..

[32] Anders P.L., Davis E.L., McCall W.D. (2014). Dental students’ attitudes toward tobacco cessation counseling. J. Dent. Educ..

