# Supplementary material for: Dental Students’ Awareness, Preparedness and Barriers towards Managing Tobacco-Using Patients—A Cross-Sectional Study
Source: Int J Environ Res Public Health. 2019 May 27;16(10):1862. doi: 10.3390/ijerph16101862 (PMC6572687; doi:10.3390/ijerph16101862)
Supplement: Supplementary file 1 [file ijerph-16-01862-s001.pdf]

STROBE Statement—Checklist of items that should be included in reports of ***cross-sectional studies***

	Item No	Recommendation	Page
**Title and abstract**	1	(*a*) Indicate the study's design with a commonly used term in the title or the abstract	1
		(*b*) Provide in the abstract an informative and balanced summary of what was done and what was found	1
**Introduction**			
Background/rationale	2	Explain the scientific background and rationale for the investigation being reported	1-2
Objectives	3	State specific objectives, including any prespecified hypotheses	2
**Methods**			
Study design	4	Present key elements of study design early in the paper	3
Setting	5	Describe the setting, locations, and relevant dates, including periods of recruitment, exposure, follow-up, and data collection	2-3
Participants	6	(*a*) Give the eligibility criteria, and the sources and methods of selection of participants	3
Variables	7	Clearly define all outcomes, exposures, predictors, potential confounders, and effect modifiers. Give diagnostic criteria, if applicable	3
Data sources/ measurement	8*	For each variable of interest, give sources of data and details of methods of assessment (measurement). Describe comparability of assessment methods if there is more than one group	3
Bias	9	Describe any efforts to address potential sources of bias	3
Study size	10	Explain how the study size was arrived at	3
Quantitative variables	11	Explain how quantitative variables were handled in the analyses. If applicable, describe which groupings were chosen and why	3
Statistical methods	12	(*a*) Describe all statistical methods, including those used to control for confounding	3
		(*b*) Describe any methods used to examine subgroups and interactions	N/A
		(*c*) Explain how missing data were addressed	3
		(*d*) If applicable, describe analytical methods taking account of sampling strategy	N/A
		(*e*) Describe any sensitivity analyses	N/A
**Results**			
Participants	13*	(a) Report numbers of individuals at each stage of study—eg numbers potentially eligible, examined for eligibility, confirmed eligible, included in the study, completing follow-up, and analysed	3
		(b) Give reasons for non-participation at each stage	N/A
		(c) Consider use of a flow diagram	N/A
Descriptive data	14*	(a) Give characteristics of study participants (eg demographic, clinical, social) and information on exposures and potential confounders	3
		(b) Indicate number of participants with missing data for each variable of interest	4-7
Outcome data	15*	Report numbers of outcome events or summary measures	3-7
Main results	16	(*a*) Give unadjusted estimates and, if applicable, confounder-adjusted estimates and their precision (eg, 95% confidence interval). Make clear which confounders were adjusted for and why they were included	N/A

		(*b*) Report category boundaries when continuous variables were categorized	N/A
		(*c*) If relevant, consider translating estimates of relative risk into absolute risk for a meaningful time period	N/A
Other analyses	17	Report other analyses done—eg analyses of subgroups and interactions, and sensitivity analyses	N/A
**Discussion**			
Key results	18	Summarise key results with reference to study objectives	7-8
Limitations	19	Discuss limitations of the study, taking into account sources of potential bias or imprecision. Discuss both direction and magnitude of any potential bias	8-9
Interpretation	20	Give a cautious overall interpretation of results considering objectives, limitations, multiplicity of analyses, results from similar studies, and other relevant evidence	7-9
Generalisability	21	Discuss the generalisability (external validity) of the study results	8
**Other information**			
Funding	22	Give the source of funding and the role of the funders for the present study and, if applicable, for the original study on which the present article is based	N/A

*Give information separately for exposed and unexposed groups.

Note: An Explanation and Elaboration article discusses each checklist item and gives methodological background and published examples of transparent reporting. The STROBE checklist is best used in conjunction with this article (freely available on the Web sites of PLoS Medicine at http://www.plosmedicine.org/, Annals of Internal Medicine at http://www.annals.org/, and Epidemiology at http://www.epidem.com/). Information on the STROBE Initiative is available at www.strobe-statement.org.

Pilot test of the questionnaire

The questionnaire was partly based on an earlier study by Murugaboopathy et al. (2013). Five dental students, who were with clinical experience and from other English-speaking countries, were recruited for pilot test. These students were not included in the main test. Advices of English editing were collected.

In the original questionnaire, to investigate the common practice of dental students managing smoker patients, questions set in section 2 (clinical practice when treating smoker patients) asked the respondents whether they had recorded the number of pack-year in patient record, the type of tobacco used. In addition, they were asked if they had made afford to assist patients to quit tobacco use. However, in the pilot testing of the questionnaire, we found out that not all dental students had smoker patients in their patient pool. Therefore, further amendment rather than the language editing was performed. In our finalized questionnaire, the same session was divided into two sub-sections asking different questions for those students who had smoker patients and those who had not. If they had smoker patients, they were asked about their actual clinical practice. Yet for those who did not have smoker patients, they were asked what they would do if they have smoker patients.

Reference

Murugaboopathy, V.; Ankola, A.V.; Hebbal, M.; Sharma, R. Indian dental students' attitudes and practices regarding tobacco cessation counseling. J Dent Educ 2013, 77, 510–517.

Dental students' awareness, preparedness and barriers towards managing tobacco-using patients

1. Views and confidence towards practicing tobacco cessation counselling

	Strongly agree	Agree	Neutral	Disagree	Strongly disagree
I will advise patients to quit tobacco use in my future career					
I believe tobacco cessation counselling provided by dentists could assist patients to quit tobacco use					
I am confident in explaining the negative impacts of tobacco usage on oral health					
I understand tobacco has a role in aetiology of oral cancer					
I am confident in providing tobacco users with written information and self-help material to assist their quitting					
I believe Nicotine Replacement Therapy is helpful for patient to quit tobacco use					
I am knowledgeable enough to introduce Nicotine Replacement Therapy for my patients (e.g. the correct dosage, the products available, the possible side effects)					
I know the mechanism of action of Nicotine Replacement Therapy					
I am well-prepared to help patients in tobacco cessation					
I know what is tobacco cessation protocol					

2. History taking habit of students for tobacco-using patients

	Yes	No
I take tobacco usage histories from all patients		
I ask patients about their tobacco usage status at the first appointment		
I update patient's smoking history regularly throughout the whole course of treatment for every patient		
I always document patient's tobacco use history in patient folder		
I have patients who are tobacco users		
I mark down patient as a "non-tobacco user" in patient folder for non-tobacco-using patients		

3. For those who **have** tobacco-using patient(s),

I ***only*** mark down patient as a "tobacco user" in patient folder for tobacco-using patients		
I have recorded the amount of tobacco my patients used over the years (e.g. pack year)in patient folder		
I have recorded the type of tobacco in patient folder (e.g. cigarettes, cigars, e-cigarettes, etc)		
I have made effort to assist a patient to quit tobacco use		
I do active tobacco cessation counselling regularly throughout the whole course of treatment for every patient		
I have succeeded in helping a patient to reduce tobacco consumption		
I have succeeded in helping a patient to quit tobacco use completely		

4. For those who do **NOT** have patient who is tobacco user

If I have a tobacco-using patient, I will ***only*** mark down patient as a "tobacco user" in patient folder		
If I have a tobacco-using patient, I will record the amount of tobacco my patients used over the years (e.g. pack year) in patient folder		
If I have a tobacco-using patient I will record the types of tobacco in patient folder (e.g. cigarettes, cigars, e-cigarettes, etc.)		

5. I believe dentists should do the following for tobacco-using patients:

	Yes	No
No tobacco cessation counselling is needed		
Giving tobacco cessation advice verbally		
Distributing leaflets or pamphlets		
Giving out the hotline for Tobacco Control Office		
Writing a referral letter to Tobacco Control Office		
Prescribe Nicotine Replacement Therapy		

6. Before a dentist commences the following treatments for tobacco-using patients (please tick if appropriate):

	Tobacco cessation is not necessary	Tobacco Cessation Counselling should be carried out	Complete tobacco cessation must be achieved
Treatments for gingivitis			
Treatments for periodontitis			
Implant placement			
Cosmetic dental treatment (eg. Veneers, crowns at aesthetic zone)			

7. Perceived barriers in delivering tobacco cessation counselling to patients

	Strongly agree	Agree	Neutral	Disagree	Strongly disagree
I do not have sufficient skills to provide smoking cessation counselling at this stage of my training					
I cannot accurately determine patient's' smoking history without being intrusive					
I do not consider tobacco cessation counselling part of the dentist's professional role					
Giving smoking cessation counselling is not part of my role as a dental student					
Patients do not expect tobacco cessation counselling from a dental student					
Patients do not listen to dental students during tobacco cessation counselling					
I am concerned that the tobacco cessation usage message may alienate tobacco-using patients					
Giving unwanted tobacco cessation counselling may upset the dentist-patient relationship					
Many tobacco-using patients do not have the motivation to quit					
Tobacco cessation counselling is ineffective unless the patient has a related health problem					
Clinical time is limited so I tend to focus on dental treatments instead of counselling					
There is no tobacco cessation information (eg. Leaflets or pamphlets) available in the hospital which I can distribute to my patient					
There is no referral pathway for tobacco-using patients					

8. Demographic data
   - What is your gender:

     ☐ M, ☐ F
   - Regarding my family's and my tobacco use status:

	Non-smoker	Former user	Current user
I am			
My immediate family members (parents or siblings)			

Thank you for your participation in our research!
